# Effects of Dietary Multi-Strain Probiotics on Growth Performance, Antioxidant Status, Immune Response, and Intestinal Microbiota of Hybrid Groupers (*Epinephelus fuscoguttatus* ♀ *× E. lanceolatus* ♂)

**DOI:** 10.3390/microorganisms12071358

**Published:** 2024-07-02

**Authors:** Guangting Xie, Xiaoying Chen, Yuwei Feng, Zhide Yu, Qiuqin Lu, Manfeng Li, Ziqi Ye, Heizhao Lin, Wei Yu, Hu Shu

**Affiliations:** 1 School of Life Sciences, Guangzhou University, Guangzhou 510006, China; xie007576@126.com (G.X.); 13714653687@163.com (X.C.); fengyuwei99@163.com (Y.F.); 15902002970@163.com (Z.Y.); lqq3215@163.com (Q.L.); mapleiroy@163.com (M.L.); y2298543@163.com (Z.Y.); 2 Key Laboratory of South China Sea Fishery Resources Exploitation & Utilization, Ministry of Agriculture and Rural Affairs, South China Sea Fisheries Research Institute, Chinese Academy of Fishery Sciences, Guangzhou 510300, China; linheizhao@163.com; 3 Shenzhen Base of South China Sea Fisheries Research Institute, Chinese Academy of Fishery Sciences, Shenzhen 518121, China

**Keywords:** *Bacillus cereus*, *Exiguobacterium acetylicum*, compound probiotics, intestinal health, aquaculture

## Abstract

**Simple Summary:**

In aquaculture, probiotics are widely used to improve growth, digestion, immunity, and so on. Employing a combination of multiple probiotic strains to enhance host health promotion is commonly acknowledged. However, studies assessing the synergistic effects of mixed probiotic additives on hosts and comparing the properties of each strain in their composition remain unclear. Therefore, this study aims to elucidate the mechanisms underlying the effects of individual and combined endogenous probiotics on hybrid groupers. Our findings revealed that the addition of mixed probiotics (*Bacillus cereus* and *Exiguobacterium acetylicum*) effectively enhances the growth performance, immune response, and intestinal health of hybrid groupers. Therefore, we recommend selecting combination strains of endogenous probiotics for optimal growth and immunity in hybrid groupers.

**Abstract:**

This study aims to examine the effects of the mixture of *Bacillus cereus* G1–11 and *Exiguobacterium acetylicum* G1–33, isolated from the gut of hybrid groupers (*Epinephelus fuscoguttatus* ♀ × *E. lanceolatus* ♂), on the host. The hybrid groupers were divided into a control (C, without any probiotics), *B. cereus* (BC, 10^10^ cfu/g), *E. acetylicum* (EA, 10^8^ cfu/g), compound (mix, a 1:1 mixture of *B. cereus* and *E. acetylicum*), and positive reference group (P, *Lactobacillus acidophilus*, 5 × 10^8^ cfu/L). Each group had four replicates, with 30 fish per replicate (53.30 ± 0.50 g), and were fed for 60 days. The results showed that adding probiotics to the feed significantly improved the weight gain, weight growth rate, specific growth rate, and digestive enzyme activities of hybrid groupers compared to the C group. The compound group was the most significant. In addition, composite probiotics added to feed significantly upregulated the expression levels of several growth-related genes in the liver and muscles. The activities of alkaline phosphatase, catalase, glutathione peroxidase, glutathione transferase, lysozyme, and total antioxidant capacity in the serum and liver were significantly influenced through mixed probiotic feeding. Moreover, the expression levels of several immune-related genes in the liver, spleen, and head kidney were significantly enhanced by adding single and mixed probiotics to feed, with the synergy of mixed probiotics being the best. An analysis of the gut microbiota showed that adding composite bacteria enhanced the richness and diversity of the gut microbiota, significantly increasing the relative abundance of potential probiotics (*Cetobacterium* and *Microbacterium*) while decreasing the presence of potential pathogens (*Mycoplasma*). Overall, our findings highlighted the efficacy of mixed probiotics (*B. cereus* and *E. acetylicum*) in enhancing growth performance, nutritional value of hybrid grouper feed, antioxidant capacity, immune response, and intestinal health, in finding the best combination of functional feed additives.

## 1. Introduction

The intensification and rapid expansion of aquaculture have led to severe stress on fish in intensive farming, potentially increasing their susceptibility to pathogens and reducing immune function [1]. This phenomenon has fueled the rapid development of fish pathogens in intensive rearing modes, leading to high mortality rates and economic losses [2]. Additionally, the widespread use of antibiotics in aquaculture has exacerbated the susceptibility of aquatic animals to pathogenic microorganisms and increased resistance to pathogenic bacteria [3]. Consequently, probiotics have emerged as a promising eco-friendly alternative to antibiotics in aquatic settings, garnering significant attention for their potential contributions to the sustainable development of the aquaculture industry [4].

Probiotics serve as common additives in aquaculture to enhance growth performance, digestion, absorption, immune responses, antioxidant levels, and intestinal barrier function [5,6]. Most probiotics used in aquaculture are commercial probiotics such as *Bacillus* and *Lactobacillus* [7]; however, the precise mechanisms by which exogenous probiotics colonize hosts and exert their effects remain unclear [8]. Endogenous probiotics, isolated from the gut of aquatic animals, have a superior ability to compete with gut microbes and effectively colonize, multiply, and persist within the gut of the host [9].

In vitro studies have revealed that *Bacillus cereus* and *Exiguobacterium acetylicum*, isolated from the gut of hybrid groupers, exhibit various probiotic traits, including acid and bile tolerance, epithelial adhesion, and antioxidant activity, and can compete with and inhibit pathogens [10]. Studies have reported the promoting effects of separately supplementing fish diets with *B. cereus* and *E. acetylicum*. When used as a feed additive, *B. cereus* can enhance fish growth performance, immune and antioxidant functions, and intestinal immune status [11,12]. Similarly, incorporating *E. acetylicum* into feed can augment the innate immunity of *Carassius auratus* and its resistance to *Aeromonas hydrophila* infection [13]. However, owing to variations in environmental conditions and host physiology, a single type of probiotic cannot meet the needs of all hosts [14]. The practice of employing a combination of multiple probiotic strains to complement or enhance host health promotion by a single strain is commonly acknowledged [5]. A diet containing multiple strains of probiotics has better disease protection and immunostimulatory effects than supplementation with a single probiotic [15]. Research on the red sea bream (*Pagrus major*) indicates that mixed probiotic supplements have a more significant influence on growth and immunity than those of a single strain [16]. Researchers are increasingly concerned about the application of multi-strain probiotics as a functional feed; however, studies evaluating the synergistic effects of mixed probiotic additives on hosts, and comparing the characteristics of multi-strain probiotics with those of each single strain in their composition, remain unclear [16]. Without such comparisons, assessing whether mixed probiotics are more effective than single strains and determining the optimal combination of beneficial bacteria to improve the diverse performance of aquatic animals becomes challenging. Therefore, this study aims to investigate the effects of the intestinal isolates of hybrid groupers (*B. cereus* and *E. acetylicum*) on growth performance, intestinal digestion and absorption, expression of growth-related genes, antioxidant status, immune response, and intestinal microbiota. Individual and combined applications are explored to identify the best combination of functional feed supplements.

## 2. Materials and Methods

### 2.1. Experimental Fish and Feeding Conditions

The hybrid groupers used in this study were obtained from the Guangdong Marine Fisheries Experimental Center. Before the experiments, the fish were fed a control diet for 2 weeks to acclimatize. Overall, 600 healthy, disease-free, and immature young hybrid groupers, all of the same age (53.30 ± 0.50 g), were selected and allocated to 20 water tanks (170 L), with 30 fish per tank. These tanks were connected to an open circulation system (salinity of 24–30‰, temperature of 28 ± 2 °C). The experiments were divided into five groups, namely the control (C, without any probiotics), *B. cereus* (BC), *E. acetylicum* (EA), mixed probiotic (mix), and positive control (P), with four parallel samples in each group.

### 2.2. Preparation of Probiotics and Feed

*B. cereus* G1–11 and *E. acetylicum* G1–33, previously isolated from the gut of hybrid groupers, were cultured in trypsin soybean soup (TSB, HuanKai, Guangdong, China) at 37 °C for 24 h and then stored in 20% (*v*/*v*) glycerol at −80 °C [10]. For the experiments, the isolated strains were inoculated into TSB (HuanKai, Guangdong, China) and then incubated at 37 °C for 24 h. The bacterial cultures were then centrifuged at 3000× *g* for 15 min at 4 °C. The bacterial sediment was washed thrice with sterile phosphate-buffered saline (Sangon, Shanhai, China) and resuspended in PBS. The concentrations of *B. cereus* and *E. acetylicum* were adjusted to 10^10^ cfu/g and 10^8^ cfu/g, respectively. The two strains were mixed in a 1:1 ratio at the final concentration to form a multi-strain probiotic formulation. The C group did not contain a bacterial suspension but contained the same volume of PBS. The commercial bacterium *Lactobacillus acidophilus* (5 × 10^8^ cfu/L, South China Sea Fisheries Research Institute, Chinese Academy of Fishery Sciences) served as the positive control [17]. The basic diet used in this study was a commercial feed with a detailed composition provided in Table 1. Following the method described by Wu et al. [18], the adjusted bacterial suspension was added to the basal diet C, 5 × 10^8^ cfu/L *L. acidophilus* to P, 10^10^ cfu/g to *B. cereus*, and 10^8^ cfu/g to EA or mix. Feeds containing probiotics were dried at low temperatures and stored in sealed plastic bags at 4 °C until subsequent use. The experimental diets were prepared every 3 days.

### 2.3. Sample Collection

After measuring growth performance, three fish were randomly selected from each tank and anesthetized with 100 mg/L eugenol (Sangon, Shanghai, China). Using a sterile dissection tool, the abdominal cavity of the hybrid grouper was opened and excess attached tissue was removed. The liver, spleen, head kidney, muscle, intestines, and intestinal contents were collected in sterile centrifuge tubes, rapidly frozen in liquid nitrogen, and then stored at −80 °C for further analysis. Blood was collected from the tail vein of the hybrid groupers and left to stand for 3 h at 4 °C, and serum was obtained through centrifugation (7100× *g*, 15 min, 4 °C), then stored at 20 °C until used for biochemical parameters determination.

### 2.4. Growth Performance

The initial weight of the hybrid groupers was recorded before the experiment, and after 60 days of feeding, the final weight was recorded. The fish were fasted for 24 h before weighing. The growth performance of the hybrid groupers was evaluated using the following formula:

Weight gain (WG, g) = final body weight − initial body weight.

Weight gain rate (WGR, %) = 100 × [(final body weight − initial body weight)/initial body weight]

Specific growth rate (SGR, %) = 100 × [ln (final body weight) − ln (initial body weight)] experimental days.

Viscerosomatic index (VSI, %) = 100 × (visceral weight/body weight)

Survival rate (SR, %) = 100 × (final number of fish/initial number of fish).

### 2.5. Determination of Enzymes Related to Digestion and Immune Function

Digestive, immune, and antioxidant enzyme activities were measured as described previously [6]. The liver and intestinal samples were weighed and homogenized in 0.85% physiological saline using a tissue crusher at a weight (g) to volume (mL) ratio of 1:9, resulting in a 10% tissue homogenate. The homogenates were then centrifuged at 1300× *g* for 10 min at 4 °C, and the supernatants were collected for analysis. The intestinal tract was tested using amylase (AMS), lipase (LPS), and pepsin assay kits (Nanjing Jiancheng Biotechnology Co., Ltd., Nanjing, China). Serum and liver samples were evaluated using acid phosphatase (ACP), alkaline phosphatase (AKP), lysozyme (LZM), total antioxidant capacity (T-AOC), catalase (CAT), glutathione S-transferase (GSH-ST), and glutathione peroxidase (GSH-PX) assays (Nanjing Jiancheng Biotechnology Co., Ltd., Nanjing, China).

### 2.6. Gene Expression Detection

Total RNA was extracted from the liver, muscle, spleen, and head kidney using the TRIZOL reagent (Vazyme, Nanjing, China). The concentration, purity, and integrity of the total RNA were determined using a microplate photometer (BioTek, Vinusky, VT, USA) and agarose gel electrophoresis. cDNA synthesis was performed using HiScript^®^III RT SuperMix for qPCR (+gDNA wiper) (Vazyme, Nanjing, China) and stored at −80 °C until use. The expression levels of genes in different tissues were examined using real-time qPCR with the LightCycler^®^ 480 instrument II (Roche, Basel, Switzerland). A 20 μL qPCR reaction system was prepared in a 96-well plate containing 10 μL of SYBR qPCR Master Mix (Vazyme, Nanjing, China), 2 μL of cDNA template, 0.4 μL of upstream and downstream primers, and 7.2 μL of ddH_2_O. The cycling parameters included 30 s at 95 °C, followed by 40 cycles of 10 s at 95 °C, 30 s at 60 °C, then 15 s at 95 °C, and 1 min at 60 °C. The relative quantitation of gene expression was analyzed using 2^−ΔΔCt^ [19]. Table 2 provides the specific qPCR primer details.

### 2.7. Analysis of Intestinal Microbial Community

Four fish were randomly chosen from each group for intestinal microflora analysis. Total bacterial DNA was extracted from the gut samples of hybrid groupers using an E.Z.N.A.^®^ DNA stool kit (Omega, Norcross, GA, USA) according to the instructions of the manufacturer. The concentration and integrity of the extracted DNA were determined using a Nanodrop 2000 spectrophotometer (Thermo Fisher Scientific, Waltham, MA, USA) and 1% agarose gel electrophoresis. DNA samples underwent PCR amplification using primers 515F (5-GTGCCAGCMGCCGCGGTAA-3) and 907R (5-CCGTCAATTCMTTTRAGTTT-3) for the V4–V5 region of the 16S rRNA gene. PCR products were purified using a UNIQ-10 PCR Purification Kit (Majorbio, Shanghai, China) to obtain community libraries. The amplicons were sequenced on an Illumina MiSeq platform (Illumina, San Diego, CA, USA) following the standard protocol. Paired-end reads were assigned to each sample using a single unique barcode. The original FASTQ files underwent quality filtering using Trimmomatic and were connected to paired-end reads using FLASH (V1.2.7) [30]. High-quality sequences were analyzed using the QIIME and UPARSE pipelines [31]. The UPARSE pipeline was used to cluster sequences into operational classification units (OTUs) with 97% similarity [32]. Representative OTU sequences in the SILVA database (Release 132, https://www.arb-silva.de/documentation/release-132/, accessed on 31 May 2024) were used to obtain the classification information. The α-diversity index (CHAO1, Shannon) was calculated using QIIME. R software (V2.15.3) facilitated the analysis and visualization of differences in the α-diversity index between groups. β-diversity analysis was conducted using principal coordinate analysis (PCoA) based on the Bray–Curtis (BC) diversity in classification group distance. Pearson’s correlation analysis was employed to investigate the relationships between growth parameters, immune and antioxidant parameters, and the relative abundance of dominant species at the phylum and genus levels.

### 2.8. Statistical Analysis

All data were expressed as mean ± SE (standard error). Significant differences were determined using one-way analysis of variance (ANOVA) and Tukey’s HSD test (*p* < 0.05) in SPSS Statistics 26.0 (IBM Inc., Chicago, IL, USA). Graphs were generated using R software (V2.15.3) with reshape2, tidyverse, and ggplot2.

## 3. Results

### 3.1. Growth Performance and Digestive Enzyme Activity

**Figure 1:**
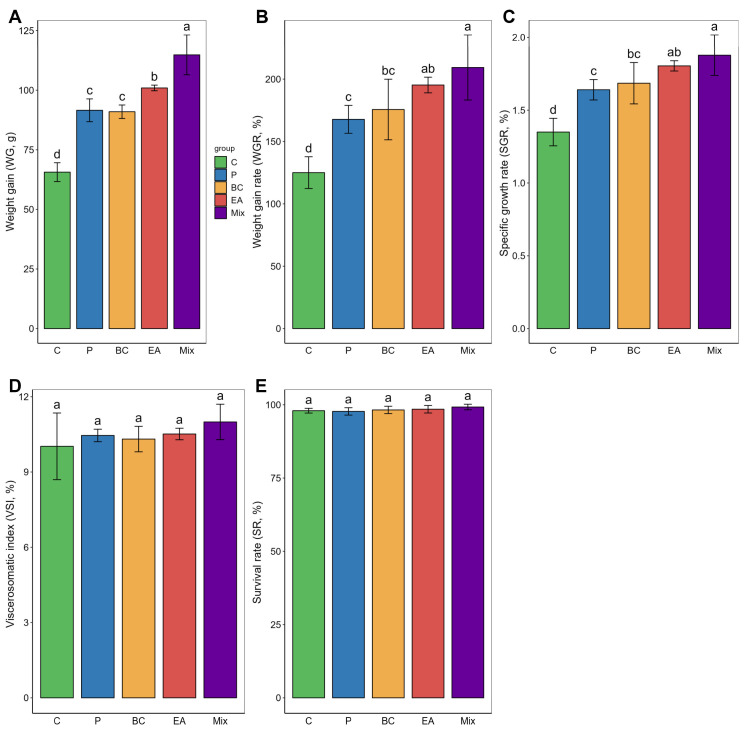
Effect of feed supplementation with single- and multiple-strain probiotics on growth performance and survival rate of hybrid groupers (*Epinephelus fuscoguttatus* ♀ × *E. lanceolatus* ♂). (**A**) Weight gain; (**B**) Weight gain rate; (**C**) Specific growth rate; (**D**) Viscerosomatic index; (**E**) Survival rate. Each value in the graph represents the mean ± SE (*n* = 4) with different letters representing significant differences (*p* < 0.05).

**Figure 2:**
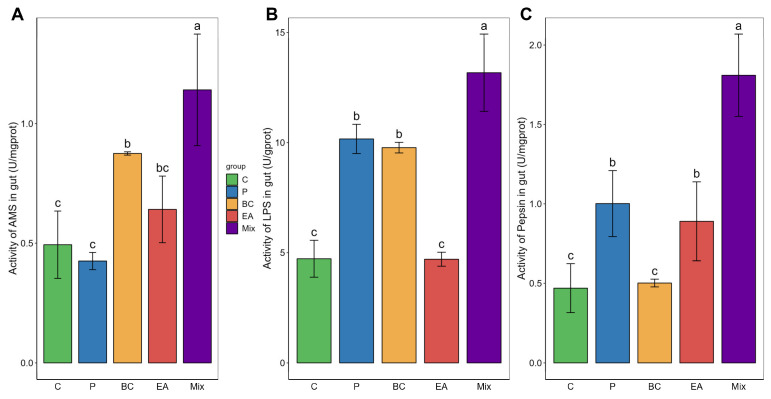
Effect of single- and multiple-strain probiotics on intestinal digestive enzyme activity in hybrid groupers (*Epinephelus fuscoguttatus* ♀ × *E. lanceolatus* ♂). (**A**) amylase activity; (**B**) lipase activity; and (**C**) pepsin activity. Each value in the figure represents the mean ± SE (*n* = 4), and the different letters represent significant differences (*p* < 0.05).

The probiotic-rich group showed significantly improved growth performance in terms of weight gain (WG), growth rate (WGR), and specific growth rate (SGR) compared to the C group after 60 days of feeding (Figure 1). The WG of the mixed group was significantly higher than that of the other groups (*p* < 0.05). In the single-bacteria groups, the WG, WGR, and SGR of the EA group were significantly higher than those of the P group (*p* < 0.05). However, the viscera-to-body ratio (VSI) among the groups was similar, with no statistically significant differences observed (*p* > 0.05). In addition, there was no significant difference among the groups regarding survival rate (*p* > 0.05). These results prompted further investigation into the activity of digestive enzymes in the guts of the hybrid groupers (Figure 2). The activities of amylase and lipase in the BC group were significantly higher than those in the C group (*p* < 0.05). The pepsin activity of the EA group was significantly higher than that of the C group (*p* < 0.05). However, the activity of these enzymes was highest in the mixed group. The activities of amylase, lipase, and pepsin in the mixed group were significantly higher than those in the C and P groups (*p* < 0.05).

### 3.2. mRNA Expression Levels of Growth-Related Genes

**Figure 3:**
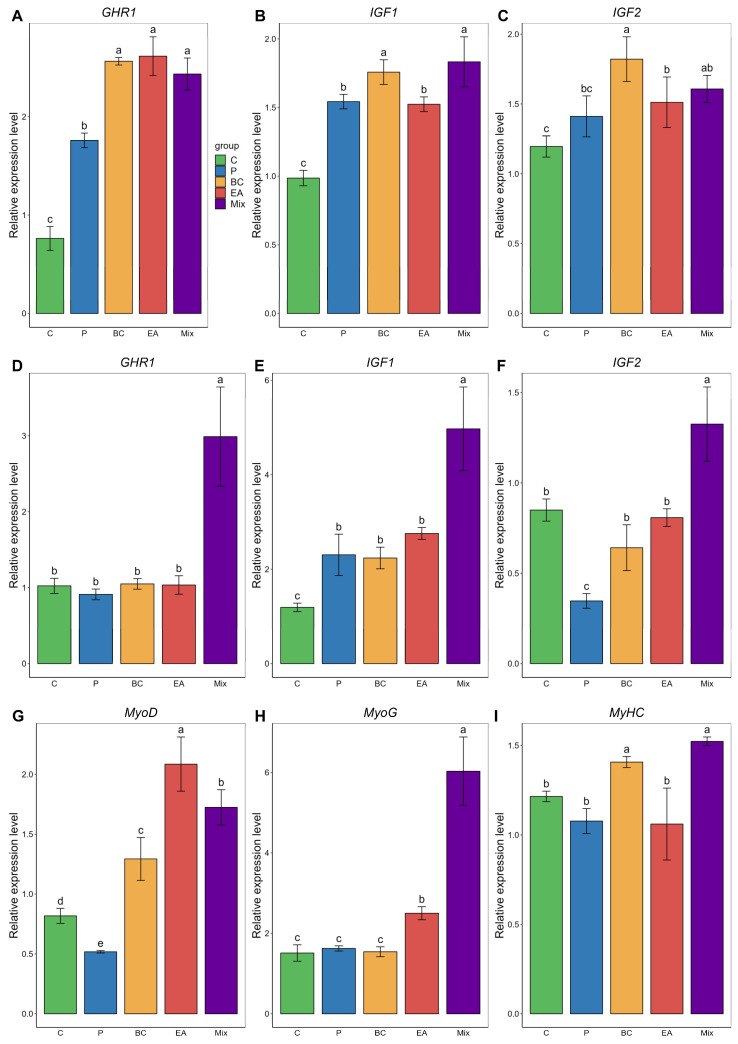
Relative expression of growth-related genes in liver (**A**–**C**) and muscle (**D**–**I**) of hybrid groupers (*Epinephelus fuscoguttatus* ♀ × *E. lanceolatus* ♂) fed with single and multiple probiotics. *GHR1*: growth hormone receptor 1, *IGF-1*: insulin-like growth factor-1, *IGF-2*: insulin-like growth factor-2, *MyoD*: muscle differentiation factor, *MyoG*: muscle differentiation factor, *MyHC*: myosin heavy chain. Each value in the figure represents the mean ± SE (*n* = 4), and the different letters represent significant differences (*p* < 0.05).

Figure 3 shows the mRNA expression levels of growth-related genes. In the liver, the addition of probiotics to the diet significantly increased *GHR1*, *IGF1*, and *IGF2* expression levels compared with the C group (Figure 3A). In the muscle, compared with the C group, the BC group exhibited significantly upregulated expression of *IGF1*, *MyoD*, and *MyHC*, whereas the EA group showed significantly increased expression levels of *IGF1* and *MyoG* (Figure 3B). However, the expression levels of *GHR1*, *IGF1*, *IGF2*, *MyoG*, and *MyHC* in the mixed group were significantly higher than those in the control and single-strain groups (*p* < 0.05).

### 3.3. Immune Response and Antioxidant Activity

**Figure 4:**
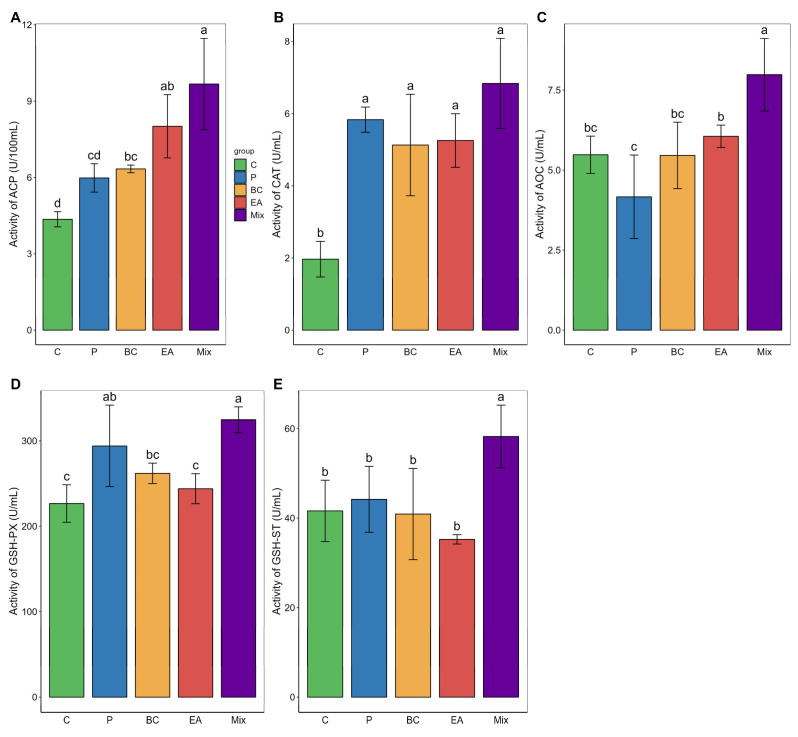
Effects of single and multiple probiotics on innate immune response and antioxidant function of serum in hybrid groupers (*Epinephelus fuscoguttatus* ♀ × *E. lanceolatus* ♂). (**A**) acid phosphatase (ACP); (**B**) catalase (CAT); (**C**) total antioxidant capacity (T-AOC); (**D**) glutathione peroxidase (GSH-PX); (**E**) glutathione S-transferase (GSH-ST). Each value in the figure represents the mean ± SE (*n* = 4), and the different letters represent significant differences (*p* < 0.05).

**Figure 5:**
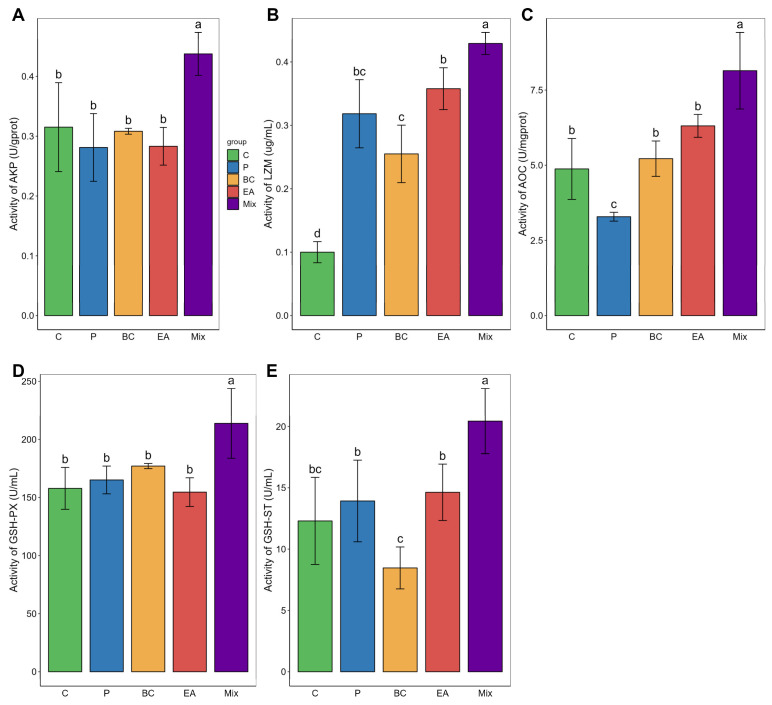
Effects of single and multiple probiotics on innate immune response and antioxidant function of liver in hybrid groupers (*Epinephelus fuscoguttatus* ♀ × *E. lanceolatus* ♂). (**A**) alkaline phosphatase (AKP); (**B**) lysozyme (LZM); (**C**) total antioxidant capacity (T-AOC); (**D**) glutathione peroxidase (GSH-PX); (**E**) glutathione S-transferase (GSH-ST). Each value in the figure represents the mean ± SE (*n* = 4), and the different letters represent significant differences (*p* < 0.05).

The immune response and antioxidant enzyme activity in the serum of the mixed group were significantly higher than those in the C group (*p* < 0.05) (Figure 4). The GSH-ST activity of the mixed group was significantly higher than that of the single-bacteria group (*p* < 0.05). Similarly, adding probiotics to the feed significantly elevated the activities of LZM, AOC, and GSH-ST in the livers of the mixed group (*p* < 0.05) (Figure 5). The activities of AKP, AOC, GSH-PX, and GSH-ST in the mixed group were significantly higher than those in the single-bacteria group (*p* < 0.05).

### 3.4. mRNA Expression Levels of Immune and Antioxidant Genes

**Figure 6:**
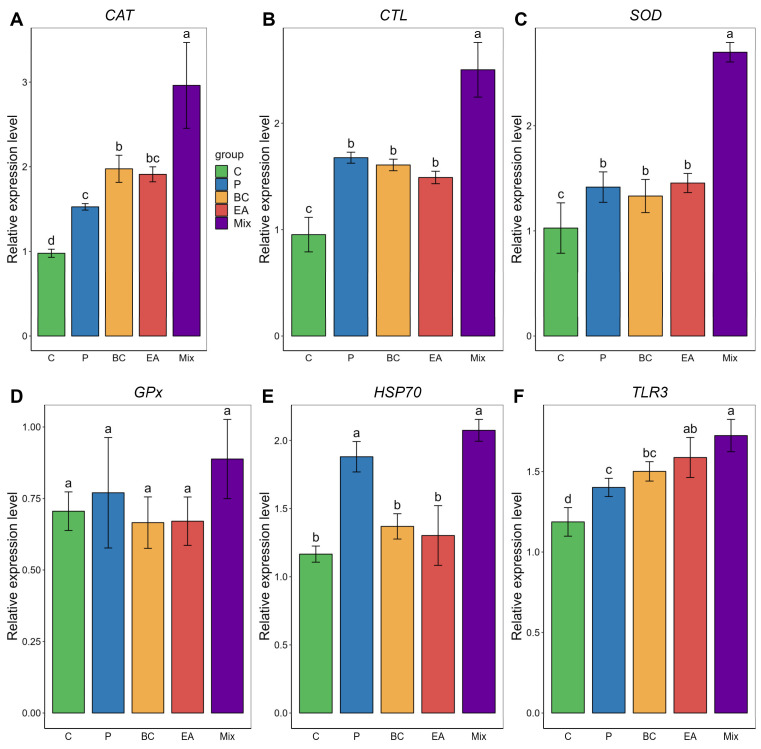
Relative expression of immune-related genes in liver of hybrid groupers (*Epinephelus fuscoguttatus* ♀ × *E. lanceolatus* ♂) fed with single and multiple probiotics. (**A**) *CAT*: catalase, (**B**) *CTL*: cytotoxic T lymphocytes, (**C**) *SOD:* superoxide dismutase, (**D**) *GPx*: glutathione peroxidase, (**E**) *HSP70*: heat shock protein, (**F**) *TLR 3*: toll-like receptor 3. Each value in the figure represents the mean ± SE (*n* = 4), and the different letters represent significant differences (*p* < 0.05).

In the liver, the expression levels of *CAT, CTL, SOD,* and *TLR3* were significantly higher in the BC and EA groups than those in the C group (Figure 6). The mixed group showed significantly upregulated expression of *CAT, CTL* and *SOD* compared to the other groups (*p* < 0.05). The *HSP70* expression level in the mixed group was significantly higher than that in the single-bacteria groups (BC and EA), but the difference was not as significant as that in the P group. *TLR3* expression levels in the mixed group were significantly higher than those in the C, P, and BC groups; however, the difference was not significant compared with those in the EA group. There was no significant difference in GPX expression levels among the groups (*p* > 0.05).

**Figure 7:**
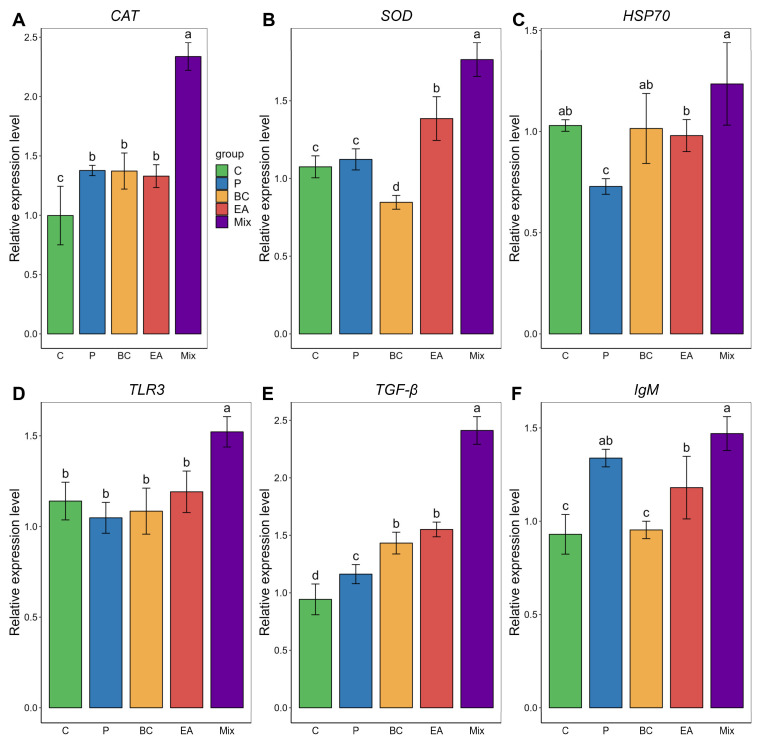
Relative expression of immune-related genes in head kidney of hybrid groupers (*Epinephelus fuscoguttatus* ♀ × *E. lanceolatus* ♂) fed with single and multiple probiotics. (**A**) *CAT:* catalase, (**B**) *SOD*: superoxide dismutase, (**C**) *HSP70:* heat shock protein, (**D**) *TLR 3*: toll-like receptor 3, (**E**) *TGF-β*: transforming growth factor-β, (**F**) *IgM*: immunoglobulin M. Each value in the figure represents the mean ± SE (*n* = 4), and the different letters represent significant differences (*p* < 0.05).

In the head kidney, the expression levels of *CAT* and *TGF-β* in the BC and EA groups were significantly higher than in the C group (*p* < 0.05) (Figure 7). The expression levels of *CAT*, *SOD*, *TLR3*, and *TGF-β* were highest in the mixed group (*p* < 0.05). The expression level of immunoglobulin M (*IgM*) in the mixed group was significantly higher than that of the C, BC, and EA groups; however, no significant difference was observed between the mixed and P groups.

**Figure 8:**
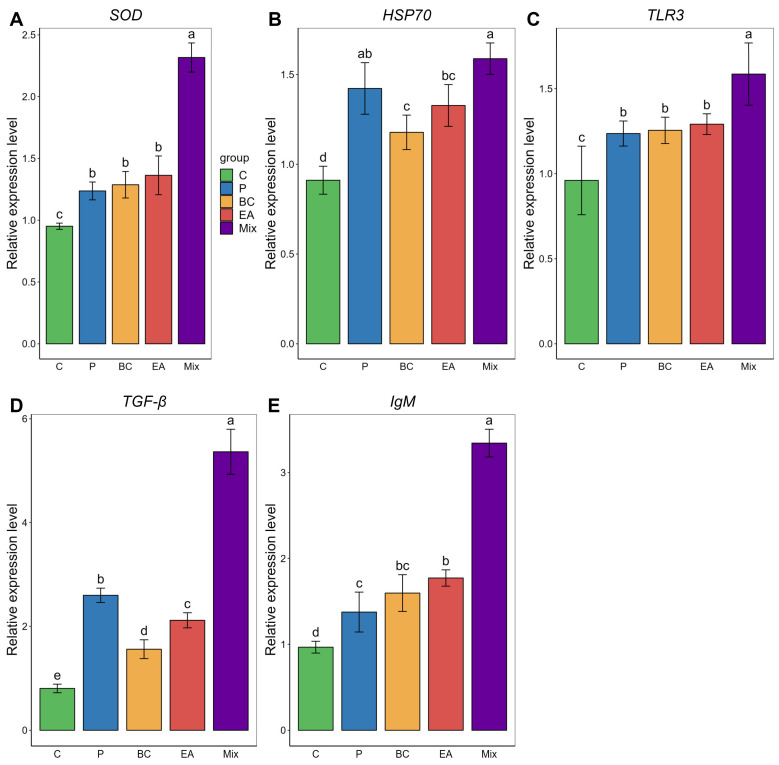
Relative expression of immune-related genes in spleen of hybrid groupers (*Epinephelus fuscoguttatus* ♀ × *E. lanceolatus* ♂) fed with single and multiple probiotics. (**A**) *SOD*: superoxide dismutase, (**B**) *HSP70*: heat shock protein, (**C**) *TLR 3*: toll-like receptor 3, (**D**) *TGF-β*: transforming growth factor-β, (**E**) *IgM*: immunoglobulin M. Each value in the figure represents the mean ± SE (*n* = 4), and the different letters represent significant differences (*p* < 0.05).

Similarly, in the spleen, the levels of *SOD*, *HSP70*, *TLR3*, *TGF-β*, and *IgM* were significantly higher in the BC and EA groups than in the control group (Figure 8). However, the expression levels of these immune-related genes in the mixed group were significantly higher than in the BC, EA, and P groups (*p* < 0.05). The *HSP70* expression level in the mixed group was significantly higher than in the BC and EA groups, but no significant difference was observed compared to the P group.

### 3.5. Effect of Probiotic Treatment on the Intestinal Microbiota of the Hybrid Groupers

**Figure 9:**
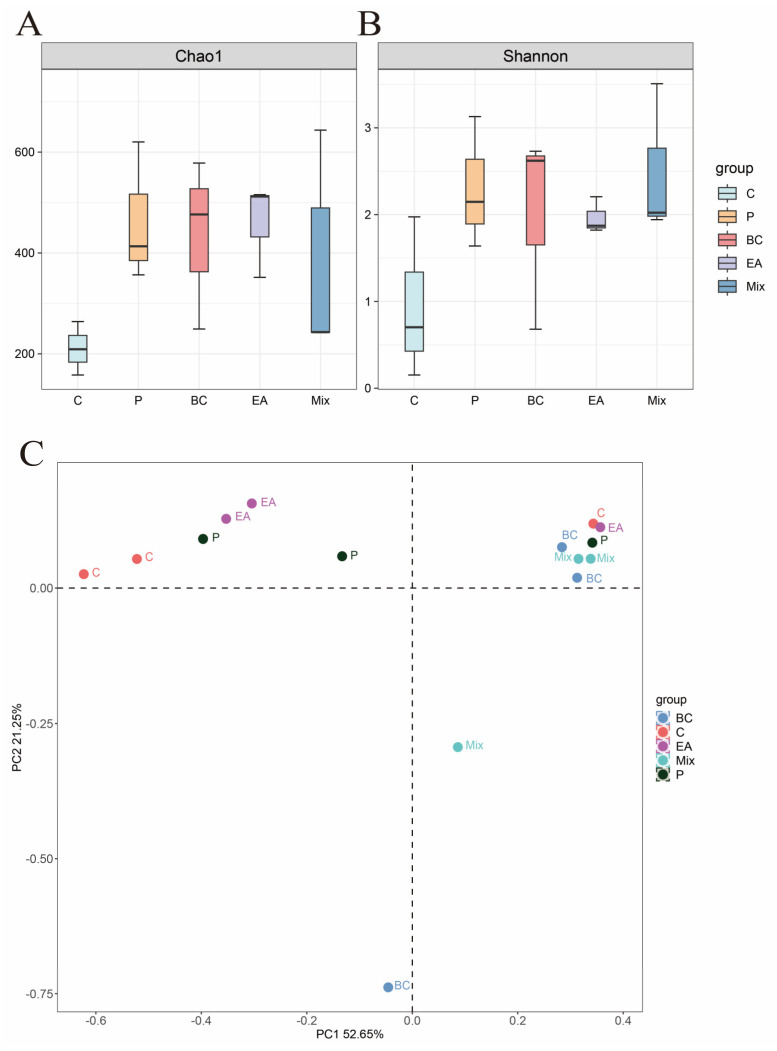
Alpha and Beta diversity analysis of intestinal microbiota of hybrid groupers (*Epinephelus fuscoguttatus* ♀ × *E. lanceolatus* ♂) fed with single and multiple probiotics. (**A**) Chao1 index; (**B**) Shannon index; (**C**) principal coordinate analysis (PCoA).

**Figure 10:**
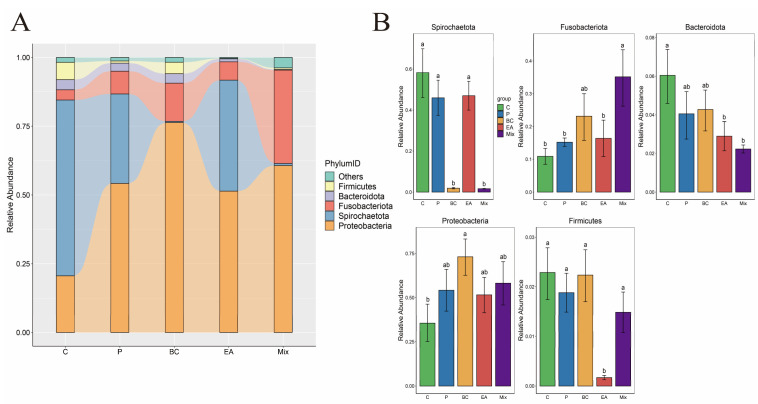
Effect of dietary supplementation of single and multiple strains of probiotics on bacterial community composition at the phylum level in the gut of the hybrid groupers (*Epinephelus fuscoguttatus* ♀ × *E. lanceolatus* ♂). (**A**) The relative abundance of microbial community at the phylum level; (**B**) the relative abundance of microbial communities in significantly changing phylum. Each value in the figure represents the mean ± SE (*n* = 4), and the different letters represent significant differences (*p* < 0.05).

**Figure 11:**
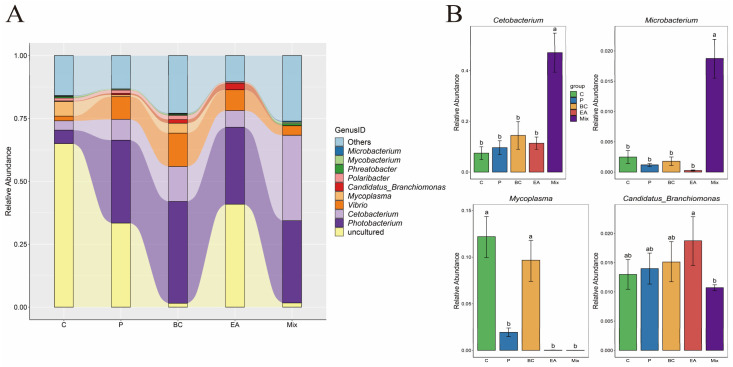
Effect of dietary supplementation of single and multiple strains of probiotics on bacterial community composition at the genus level in the gut of the hybrid groupers (*Epinephelus fuscoguttatus* ♀ × *E. lanceolatus* ♂). (**A**) The relative abundance of the microbial community at the genus level; (**B**) the relative abundance of microbial communities in significantly changing genus. Each value in the figure represents the mean ± SE (*n* = 4), and the different letters represent significant differences (*p* < 0.05).

The effect of probiotic supplementation to feed on the gut microbiota diversity of hybrid groupers was explored in this study (Figure 9). The results showed that the addition of probiotics to the feed increased the richness and diversity of gut microbiota in the hybrid groupers compared to the C group, but the effect was not significant (*p* > 0.05) (Figure 9A). Additionally, PCoA revealed no significant differences between the control and treatment groups (Figure 9B). At the phylum level, *Proteobacteria* dominated, followed by *Spirochaetota*, *Fusobacteria*, *Bacteroidota*, and *Firmicutes* (Figure 10A). The relative abundances of *Spirochaetota* and *Bacteroidota* in the mixed group were significantly lower than in the C group (*p* < 0.05) (Figure 10B). The relative abundance of *Fusobacteria* in the mixed group was significantly higher than that in the C, P, and EA groups (*p* < 0.05). At the genus level, the relative abundances of the genera *Cetobacterium* and *Microbacterium* in the mixed group were significantly higher than those in the other groups (*p* < 0.05) (Figure 11B). The addition of probiotics to the feed significantly reduced the relative abundance of *Mycoplasma* (*p* < 0.05). However, the relative abundance of *Candidatus Branchiomonas* in the mixed group was lower than in the EA group (*p* > 0.05).

**Figure 12:**
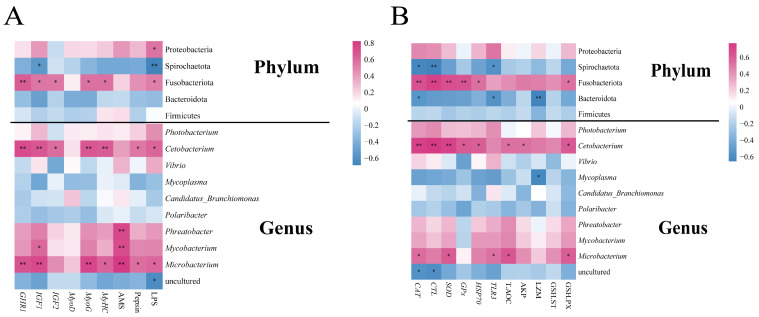
The heatmap shows the Pearson correlation between bacterial community composition and growth parameters (**A**), as well as immune and antioxidant parameters (**B**) in the gut of hybrid groupers (*Epinephelus fuscoguttatus* ♀ × *E. lanceolatus* ♂) fed with single and multiple strains of probiotics. * and ** indicate statistical differences at the *p* < 0.05 and *p* < 0.01 levels, respectively.

To explore the relationship between the gut microbiota and growth parameters, a Pearson correlation analysis was performed (Figure 12A). At the phylum level, *Fusobacteria* exhibited significant positive correlations with *GHR1*, *IGF1*, *IGF2*, *MyoG*, *MyHC*, and LPS (*p* < 0.05, *p* < 0.01), while displaying significant negative correlations with *Bacteroidota* (*p* < 0.05, or *p* < 0.01). At the genus level, most growth parameters were significantly positively correlated with *Cetobacterium* and *Microbacterium* (*p* < 0.05, *p* < 0.01, respectively), whereas AMS was significantly positively correlated with *Phreatobacter, Mycobacterium,* and *Microbacterium* (*p* < 0.01). Furthermore, a correlation analysis was employed to explore the relationship between intestinal microbiota and immune parameters (Figure 12B). At the phylum level, *Fusobacteria* showed significant positive associations with *CAT*, *CTL*, *SOD*, *GPx*, *HSP 70,* and GSH-PX (*p* < 0.05, *p* < 0.01), whereas *Spirochaetota* and *Bacteroidota* displayed significant negative correlations with *CAT*, *CTL*, *TLR3*, and LZM (*p* < 0.05 or *p* < 0.01). At the genus level, most immune and antioxidant parameters exhibited significantly positive associations with *Cetobacterium* and *Microbacterium* (*p* < 0.05 or *p* < 0.01, respectively) and similar associations with *Mycoplasma* (*p* < 0.05).

## 4. Discussion

Probiotics, sought after as eco-friendly alternatives to antibiotics, are increasingly vital in sustainable aquaculture. Studies have demonstrated the ability of probiotics to bolster host growth, enhance innate immunity, and regulate the gut microbiota [33,34,35,36]. In aquaculture, mixed probiotics exhibit higher efficacy than single strains due to their synergistic effects [5]. Mixing multiple probiotic strains can supplement or amplify the beneficial effects of a single strain on host health [15]. Studies have shown that the addition of *B. cereus* and *E. acetylicum* can enhance the growth performance and immune responses of aquatic animals [13,33]. However, this study is the first to evaluate their synergistic effects on hybrid groupers. This study confirmed that *B. cereus* G1–11 and *E. acetylicum* G1–33, sourced from the gut of hybrid groupers, significantly improved growth performance, immune response, and intestinal health at optimal concentrations. However, when the two were mixed in equal proportions, a “1 + 1 > 2” effect was achieved.

Probiotics serve as widely utilized nutritional supplements for aquatic animals because they enhance WG, WGR, and SGR [37]. Generally, when incorporated into a diet, mixed strains outperform single strains in enhancing growth performance [36]. In this study, the addition of probiotics to feed significantly improved the growth performance of hybrid groupers, with the mixture yielding the most significant improvement, which was similar to the results of previous studies [11,38]. The use of probiotics can enhance the growth performance of aquatic animals, potentially attributed to their role in improving digestion mediated by digestive enzymes [39]. Probiotics not only contribute to the biosynthesis of various amino acids and vitamins but also promote nutrient absorption by augmenting the activity of intestinal digestive enzymes [10,33]. In this study, separately feeding *B. cereus* G1–11 and *E. acetylicum* G1–33 increased the activities of amylase, lipase, and pepsin. However, their combined addition significantly elevated the activities of these enzymes, consistent with the results for Nile tilapia (*Oreochromis niloticus*) [11,40].

The growth hormone (GH)–insulin-like growth factor (IGF) axis is a well-known regulator of endocrine growth in most vertebrates [41]. This axis encompasses growth hormone receptors (*GHR1* and *GHR2*) and insulin-like growth factors (*IGF1* and *IGF2*) [42]. Probiotics can improve growth by modulating the expression of growth hormones and growth factor genes [40]. In this study, the separate addition of *B. cereus* G1–11 and *E. acetylicum* G1–33 alone did not significantly affect the expression of *GHR1*, *IGF-1,* or *IGF-2*. However, the addition of the composite bacteria significantly increased their expression, providing further insights into the observed growth performance results. Similar findings have been reported for *P. Major* and *O. niloticus* [16,40].

As regulators of myogenesis, *MyoD* and *MyoG* play crucial roles in muscle cell differentiation and development [43]. Myofibrils are pivotal for muscle growth in fish, with the myosin heavy chain (*MyHC*), as the basic unit of myosin, closely related to the muscle fiber type; therefore, changes in *MyHC* expression are of great significance in analyzing fish muscle growth [44]. Previous studies primarily explored the effects of nutritional additives on fish muscle growth through the lens of muscle growth-related genes [44]. In this study, the expression levels of *MyoD*, *MyoG,* and *MyHC* significantly increased after feeding with mixed probiotics, suggesting that supplementation with probiotic mixtures can enhance muscle development and differentiation in hybrid groupers. Shadrack et al. (2022) also observed significant improvements in the expression of muscle growth-related genes in *P. Major* with mixed probiotic feeding [16].

In aquatic animals, probiotic additives can bolster innate immunity by triggering various protective mechanisms [45]. AKP serves as an effective antibacterial agent, contributing to macrophage activation [46]. ACP functions as a lysosomal enzyme, aiding in the digestion of invading organisms [47]. LZM has good antibacterial activity, effectively lysing Gram-positive and Gram-negative bacteria [48]. Various *Bacillus* strains can significantly boost the activities of AKP, ACP, and LZM, thereby improving the immune function of turbot (*Scophthalmus maximus*) [37]. In this study, single-strain feeding significantly elevated ACP levels in the serum and LZM levels in the liver, while mixed probiotic feeding resulted in the highest immune index. Previous reports indicate that mixed probiotics can more effectively improve the innate immunity of *O. niloticus* than single probiotic strains, supporting the results of the present study [40]. Similarly, dietary multi-strain probiotics enhance immune defense mechanisms in fish by enhancing the activities of ACP, AKP, and LZM [14].

Excessive levels of ROS can induce oxidative damage in the body, necessitating the removal of ROS and repair of oxidative damage to reduce the oxidative stress response in fish [24]. Previous studies have shown that *B. cereus* G1–11 and *E. acetylicum* G1–33 act as antioxidants, effectively reducing the oxidative stress responses in the host gut [10]. Fish repair oxidative damage by secreting various antioxidant enzymes [40]. SOD, CAT, AOC, GSH-PX, and GSH-ST serve as crucial indicators of antioxidant capacity in fish, and they can be used as biomarkers of oxidative stress [12,40]. The addition of different *Bacillus* species to feed significantly improves the antioxidant status of turbot (*S. maximus*), which is due to the increased activity of T-AOC and T-SOD [37]. The current study revealed that the addition of probiotics effectively enhanced the antioxidant capacity of hybrid groupers, with the synergistic effect of using multiple probiotic strains being the most significant. Findings indicate that dietary supplementation with *Bacillus* can increase the antioxidant capacity of *O. niloticus* by boosting SOD and CAT activities, especially with mixed *Bacillus* supplementation [40]. A mixture of *Lactobacillus* and *L. faecalis* can enhance the antioxidant status of Channa argus (*C. argus*) by increasing the activities of SOD, CAT, GSH-Px, and T-AOC [6]. Similar findings were reported for *Pangasianodon Hypophthalmus* and mrigal (*Cirrhinus Mrigala*) [5,12].

*IgM* plays a crucial regulatory role as an antibody in innate and adaptive immunity in fish [49]. Toll-like receptors (*TLRs*) recognize various pathogen-associated molecular patterns (PAMPs) and regulate cell synthesis and inflammatory responses [50]. The transforming growth factor, as a pleiotropic cytokine, plays a role in regulating cell proliferation and differentiation, as well as activating and deactivating immune cell functions [51]. CTL are immune molecules involved in pathogen recognition and may play a regulatory role in the immune defense mechanisms of fish [24]. *HSP70* acts as a molecular chaperone, enhancing resistance to pathogens and antioxidant activity in synergistic immunity [52]. *SOD* and *CAT* serve as the frontline defense against various oxidative stress responses in the immune system [24,40]. Studies have shown that adding *Clostridium butyricum* to diets can significantly improve the gene expression levels of *SOD, CAT*, and *GSH-Px* in hybrid groupers, thus enhancing cellular antioxidant capacity [27]. *Rummeliibacillus stabekisii* can significantly upregulate the expression levels of *TGF-β* and *HSP70*, enhancing the immune system of fish hosts [53]. The present study showed that single probiotics and multiple strains significantly upregulated the expression levels of multiple immune and antioxidant genes (*CTL*, *CAT*, *SOD*, *HSP70*, *TLR3*, *TGF-β*, and *IgM*) in the liver, spleen, and head kidney. The expression level of mixed probiotics was the highest, resulting in the most pronounced effect. Similar to our results, mixed feeding with multiple *Bacillus* strains significantly increased the *TLR-2* and *IgM* contents in *O. niloticus* [40]. Similarly, single-bacteria and mixed feeding with multiple strains significantly enhanced the expression of immune-related genes [18]. Our study revealed that single bacteria and mixed feeding of multiple strains significantly improved immune defense mechanisms and effectively bolstered antioxidant capacity in hybrid groupers, particularly when utilizing multiple-strain probiotics. However, further experiments should be conducted using pathogens such as *Vibrio*.

The gut microbiota can promote host growth, digestion and absorption, and immune responses, and is crucial for maintaining host health [6]. Previous studies have primarily focused on the effects of probiotics on the gut microbiota and the health of aquatic animals [35,39]. *Proteobacteria* and *Firmicutes* are commonly found in the gastrointestinal mucosa and contents, prevailing in species such as *Channa argus* and Turbo [6,37]. Similarly, *Proteobacteria*, *Fusobacteria*, *Firmicutes*, and *Bacteroidota* are prevalent phyla in *O. niloticus* when fed probiotics [54]. In this study, these phyla were dominant across all the treatment groups. Feeding with the mixed probiotics significantly increased *Fusobacteria* abundance while reducing spirochetes and *Bacteroidota*. *Fusobacteria,* known for producing butyrate, have shown promise as probiotics in aquaculture [55]. *Bacteroidota*, recognized as secondary pathogens, are involved in various fish diseases and can cause widespread disease outbreaks in aquatic animals [56]. A similar pattern was observed in an MA study where *B. licheniformis* LMF1 facilitated the colonization of *Proteobacteria* and *Firmicutes* in the gut of turbot (*S. maximus*) while inhibiting the *Bacteroidota* colonization [37]. At the genus level, the mixed probiotic group exhibited a significant increase in the relative abundance of *Cetobacterium* and *Microbacterium*, with a significant decrease in *Mycoplasma* abundance. In addition, the relative abundance of *Candidatus Branchiomonas* in the mixed probiotic group was lower than in the EA group. *Cetobacterium,* derived from *Fusobacteria*, is known for producing short-chain fatty acids and vitamin B-12, which are essential for maintaining intestinal integrity and promoting the metabolism of lipids, proteins, and carbohydrates [54]. Adding *Cetobacterium* to feed enhances immune response, intestinal health, and pathogen resistance [57]; therefore, *Cetobacterium* plays a pivotal regulatory role in the growth, development, and immune defense mechanisms of the host [58]. *Microbacterium,* characterized by significant heat resistance, can produce small amounts of lactic acid, creating an acidic environment in the intestine that contributes to the reduction in pathogen colonization [57]. *Candidatus Branchiomonas* is the main pathogen associated with gill disease in rainbow trout (*Oncorhynchus mykiss*) [59]. *Mycoplasma*, a pathogenic agent, is known to induce severe systemic respiratory diseases in hosts [60]. Similarly, administration of *B. subtilis* can increase the abundance of *Cetobacterium* in bullfrog guts while reducing *Mycoplasma levels* [61]. The use of *B. cereus* NY5 as a water additive also increased the relative abundance of *B. cereus* in the gut of O. *niloticus* [62].

Our findings also revealed significant positive correlations (*p* < 0.05 or *p* < 0.01) between most growth parameters and gut probiotics, such as *Fusobacteria* (phylum), *Cetobacterium* (genus), and *Microbacterium* (genus) (*p* < 0.05). Conversely, these parameters significantly negatively correlated with *Spirochaetota* (*p* < 0.05 or *p* < 0.01). Most immune and antioxidant parameters were significantly positively associated with *Fusobacteria*, *Cetobacterium,* and *Microbacterium* (*p* < 0.05, *p* < 0.01, respectively) while displaying significant negative associations with *Mycoplasma* (*p* < 0.05). Similarly, the abundance of *L. acidophilus* in the gut microbiota of Pacific white shrimp (*Litopenaeus vannamei*) is related to its growth [63]. The addition of sucrose was inversely correlated with the abundance of *Mycoplasma*, indicating that sucrose supplementation may promote the growth of shrimp by reducing pathogen adhesion and colonization [60]. Therefore, mixed probiotic feeding can increase the colonization of beneficial intestinal bacteria while reducing the colonization of pathogenic bacteria. This enhances the growth of the host, immune-related gene expression levels, and the activity of digestive and antioxidant enzymes, ultimately improving growth performance, digestive capacity, immune response, antioxidant mechanism, and intestinal barrier function.

## 5. Conclusions

In conclusion, this study demonstrates that single and mixed strains (*B. cereus* and *E. acetylicum*) isolated from the gut of hybrid groupers (*Epinephelus fuscoguttatus* ♀ × *E. lanceolatus* ♂) enhanced the growth performance, intestinal digestive function, antioxidant capacity, immune response, and intestinal health of hybrid groupers. However, the combination of the two strains at a 1:1 ratio outperformed single probiotics. Therefore, we recommend selecting combination strains of endogenous probiotics for optimal growth and immunity in hybrid groupers. Our research showed that mixed feeding with multiple strains could significantly improve the immune defense mechanism and effectively enhance the antioxidant capacity of hybrid groupers; however, further challenging experiments are warranted using pathogens such as *Vibrio*.

## Figures and Tables

**Table 1:** Composition and nutrient levels of experimental diets (g kg^−1^).

Ingredients	Diet
Fish meal	450.00
Soybean meal	130.00
Flour	237.40
Beer yeast powder	50.00
Fish oil	50.00
Soybean oil	50.00
Lecithin	10.00
Vitamin premix ^1^	2.00
Mineral premix ^2^	5.00
Choline chloride (50%)	5.00
Antioxidant	0.10
Vitamin C	0.50
Monocalcium phosphate	10.0
**Proximate Composition**	
Moisture	5.12
Crude protein	49.63
Crude lipid	14.81
Ash	10.36

^1^ Vitamin premix provides the following (mg kg^−1^ diet): vitamin A (500,000 IU) 40 mg, vitamin B_1_ 40 mg, vitamin B_2_ 93.75 mg, vitamin B_6_ 20 mg, vitamin B_12_ (1%) 45 mg, vitamin K_3_ (50%) 300 mg, inositol 400 mg, calcium pantothenate 250 mg, nicotinic acid 450 mg, folic acid 6 mg, biotin (2%) 10 mg, vitamin D_3_ (500,000 IU) 15 mg, vitamin E (50%) 300 mg, unite bran 2990.25 mg. ^2^ Mineral premix provides the following (mg kg^−1^ diet): Na_2_SeO_3_ (1%) 20 mg, CuSO_4_·5H_2_O (25%) 24 mg, FeSO_4_·H_2_O (30%) 266.65 mg, ZnSO_4_·H_2_O (34.50%) 100 mg, MnSO_4_·H_2_O (31.80%) 120 mg, Ca(IO_3_)_2_ (5%) 50 mg, CoSO_4_·7H_2_O (5%) 10 mg, zeolite power 4380.55 mg.

**Table 2:** Primers and amplification conditions used for qPCR.

Target Genes	Primers Sequences (5′–3′) for qPCR	Amplification Efficiency (%)	Amplicon Size (bp)	Accession No.	Reference
*β-actin*	F: GACATCAAGGAGAAGCTGTG	98	214	AY510710.1	[20]
	R: TGCTGTTGTAGGTGGTCTCGT				
*GHR1*	F: CACAGACTTCTATGCCCAGGT R: GTGTAGCCGCTTCCTTCAG	99	144	KR269817.1	[21]
*IGF-1*	F: GTGCGATGTGCTGTATCTCCTG R: GCCATAGCCTGTTGGTTTACTG	104	179	XM_033614181.1	[22]
*IGF-2*	F: CCGCAAAGATACGGACACCA R: GCCGACGCTATTTCCACAAC	101	146	XM_033635279.1	[22]
*MyoD*	F: GGCTCAGCAAGGTCAACGA R: CTCGATGTAGCTGATGGCGT	98	113	XM_033635008.1	[22]
*MyoG*	F: GAGGAGCACCTTGATGAACCC R: TGGGCTCACTTGAAGACGACA	102	191	XM_033625713.1	[22]
*MyHC*	F: AAGGTGTTGGCTGAGTGGAAA R: TGGATGCTCTTGCCAGTCTCA	96	221	XM_033644959.1	[22]
*CAT*	F: GCTCTATCCGCTCCTCTTCTCCTC R: GTAGTTCCTGACGACGGTGATGTG	105	115	KT884509.1	[23]
*CTL*	F: GGCAAGATGGGAGCCTAAC R: ATGCGACGAATGTCCTGAA	95	121	FJ805451.1	[24]
*SOD*	F: GTTGGAGACCTGGGAAATGTGACTG R: CCATTGAGGGTGAGCATCTTGTCC	102	83	AY735008.1	[23]
*GPx*	F: TCCTCTGTGGAAGTGGCTGA R: TCATCCAGGGGTCCGTATCT	99	132	HQ441085.1	[25]
*HSP70*	F: GTCCTGATCAAACGAAACACCA R: CACGCTCACCCTCATAAACCT	101	128	AY423555.2	[26]
*TGF-β*	F: AACATCCCGCTACCTCGCTT R: TCCGCTCATCCTCATTCCCT	94	116	XM_049576571.1	[27]
*IgM*	F: ACCGTGACCCTGACTTGCTATG R: CCCGATGGACCTGACAATAGC	95	78	GU988694.1	[28]
*TLR 3*	F: CTGGCTTACTACAACCACCCC	105	226	HQ857748.1	[29]
	R: CAAACTCCCTGCCCTCTTCA				

*GHR1*: growth hormone receptor 1, *IGF-1*: insulin-like growth factor-1, *IGF-2*: insulin-like growth factor-2, *MyoD*: muscle differentiation factor, *MyoG*: muscle differentiation factor, *MyHC*: myosin heavy chain, *CAT*: catalase, *CTL*: cytotoxic T lymphocytes, *SOD*: superoxide dismutase, *GPx*: glutathione peroxidase, *HSP70*: heat shock protein, *TGF-β:* transforming growth factor-β, *IgM*: immunoglobulin M, *TLR 3*: toll-like receptor 3.

## Data Availability

Results of all analyses are included in this published article. The datasets generated and/or analyzed during the current study are available from the corresponding author upon reasonable request.
